# Analysis of heteroplasmy in bank voles inhabiting the Chernobyl exclusion zone: A commentary on Baker et al. (2017) “Elevated mitochondrial genome variation after 50 generations of radiation exposure in a wild rodent.”

**DOI:** 10.1111/eva.12578

**Published:** 2018-01-17

**Authors:** Jenni Kesäniemi, Zbyszek Boratyński, John Danforth, Prince Itam, Toni Jernfors, Anton Lavrinienko, Tapio Mappes, Anders Pape Møller, Timothy A. Mousseau, Phillip C. Watts

**Affiliations:** ^1^ Department of Ecology and Genetics University of Oulu Oulu Finland; ^2^ CIBIO/InBIO, Research Center in Biodiversity and Genetic Resources University of Porto Vairão Portugal; ^3^ Department of Biological and Environmental Science University of Jyväskylä Jyväskylä Finland; ^4^ Ecologie Systématique Evolution Université Paris‐Sud, CNRS, AgroParisTech Université Paris‐Saclay Orsay Cedex France; ^5^ Department of Biological Sciences University of South Carolina Columbia SC USA

**Keywords:** ecological genetics, molecular evolution, population ecology

## 1 INTRODUCTION

Exposure to ionizing radiation is a well‐established cause of mutation. Given the global problem of accidental release of radionuclides into the environment (Lourenço, Mendo, & Pereira, 2016), it is essential to fully understand the genetic consequences of exposure to radionuclides. On 26 April 1986, a fire and explosion in Reactor 4 of the former nuclear power plant at Chernobyl (CNPP), Ukraine, released more than 9 million terabecquerels (TBq) of radionuclides over much (>200,000 km^2^) of Europe and eastern Russia (see reviews on the effects, e.g., Mousseau & Møller, 2012; Møller & Mousseau, 2006). The Chernobyl Exclusion Zone (CEZ) was established at about a 30‐km radius around the accident site to limit human exposure to radioactive fallout. The CEZ contains elevated levels of persistent radioisotopes, notably strontium‐90 (^90^S), caesium‐137 (^137^Cs) and plutonium‐239 (^239^Pu) that have half‐lives of 28.8, 30.2 and 24,100 years, respectively. Wildlife inhabiting the CEZ provide clear models of the biological consequences of exposure to environmental radionuclides, with many reports of elevated levels of developmental instability, genetic damage and mutation rate associated with inhabiting areas contaminated by radionuclides. Hence, a meta‐analysis revealed a strong effect of radiation upon mutation rate in organisms affected by Chernobyl fallout (data for 30 species in 45 published studies) (Møller & Mousseau, 2015). With this in mind, the report by Baker et al. (2017) in *Evolutionary Applications* of elevated levels of genetic diversity rates in bank voles inhabiting the CEZ appears consistent with the putative mutagenic effect of exposure to radionuclides.

**Figure 1:**
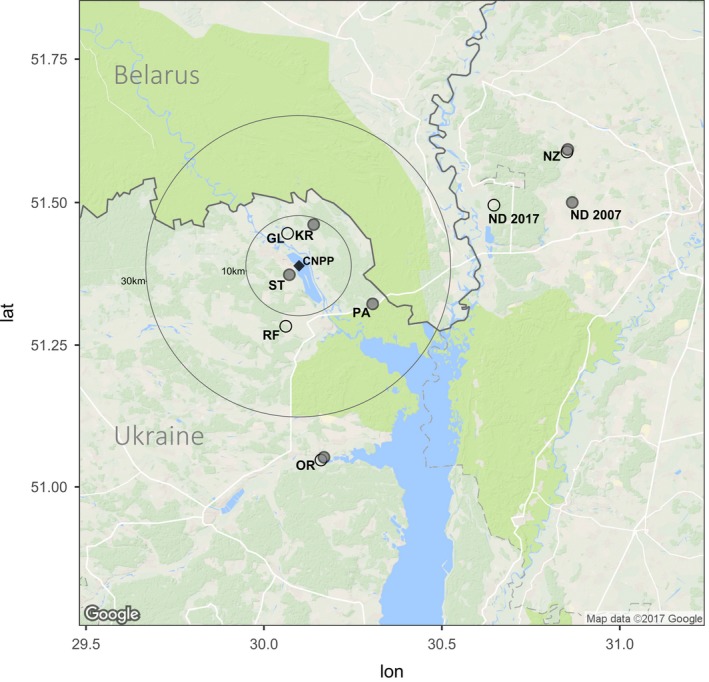
Location of sites within and outside the Chernobyl Exclusion Zone (CEZ) from which bank voles were collected, as described by Meeks et al. (2007) (filled circles) and by Baker et al. (2017) (open circles). CNPP refers to the location of the former Chernobyl Nuclear Power Plant (filled square) and circles indicate 10‐km and 30‐km radius from the CNPP. Contaminated sample sites are CL—Glyboke Lake and RF—Red Forest. Uncontaminated (references) sample sites are NZ—Nezamozhnya, ND—Nedanchychy, OR—Oranoe, PA—Paryshev, ST—Stupnikovo and KR—Krasnoye. ND 2017 refers to the Nedanchychy location reported by Baker et al. (2017), while ND 2007 refers to the location of the site used by Meeks et al. (2007): note that the coordinates for the Red Forest contaminated site in Baker et al. (2017) are ~12 km from the original Red Forest site. Map was created in R v.3.3.3 (R Development Core Team, 2014) using GGMAP v. 2.6.1 (Kahle & Wickham, 2016)

The analysis by Baker et al. (2017) is promising for two principal reasons: (i) they have data from two time points and (ii) they use next‐generation sequencing (NGS) to identify polymorphisms and thus bring studies of Chernobyl wildlife into the genomics era. Baker et al. (2017) sequenced whole mitochondrial genomes of samples of the bank vole *Myodes glareolus* to determine whether the bank voles inhabiting the CEZ have accumulated mutations as a consequence of exposure to elevated levels of radionuclides. The bank vole is a small rodent that is common in forest habitats in northern Europe. As this species is common within and around the CEZ, the bank vole has been widely studied as a model of the mammalian response to radionuclides (Boratyński, Lehmann, Mappes, Mousseau, & Møller, 2014; Chesser et al., 2000; Lehmann, Boratyński, Mappes, Mousseau, & Møller, 2016; Meeks, Chesser, Rodgers, Gaschak, & Baker, 2009; Meeks et al., 2007; Rodgers & Baker, 2000; Rodgers, Wickliffe, Phillips, Chesser, & Baker, 2001). Baker et al. (2017) found greater mitochondrial diversity in samples from two contaminated areas (Red Forest and Glyboke Lake) than in samples of bank voles from three uncontaminated (control) areas (Nedanchychy, Nezamozhnya and Oranoe) (see Figure 1 for sample locations). In the abstract, the authors state [that their data are] “*consistent with the possibility that chronic, continuous irradiation resulting from the Chernobyl disaster has produced an accelerated mutation rate in this species over the last 25 years*.” However, Baker et al., 2017 did not fully discuss three important issues relating to their data: (i) sampling, (ii) bank vole population dynamics and (iii) heteroplasmy.

## 2 SAMPLING: CORRECTION FOR VARIATION IN SAMPLE SIZES

**Figure 2:**
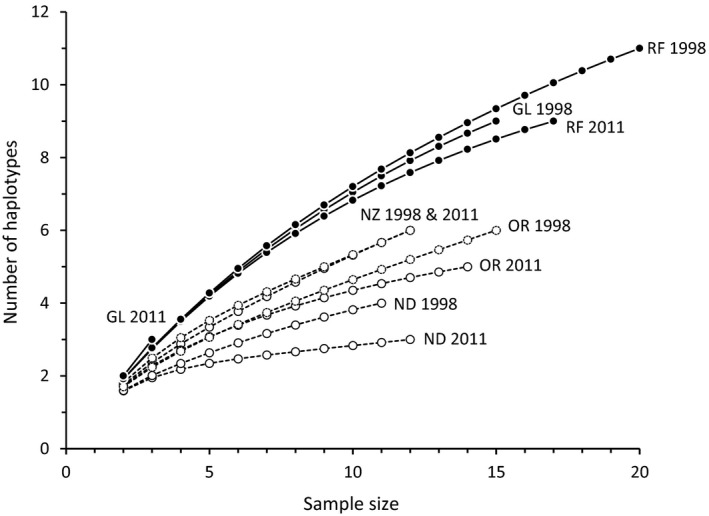
Rarefaction curves of mitochondrial genetic diversity (number of haplotypes at the ND4 locus) against sample size for ten samples of bank voles from sites that were contaminated (filled circles, solid lines) or uncontaminated (open circles, dashed lines) by environmental radioactivity. Sample codes are CL—Glyboke Lake, RF—Red Forest, NZ—Nezamozhnya, ND—Nedanchychy and OR—Oranoe

Sample sizes used by Baker et al. (2017) vary between 11 and 20 (and one sample of three bank voles at Glyboke Lake), with the two largest samples from the Red Forest (i.e., contaminated site). Baker et al. (2017) discuss possible effects of unbalanced sample size on their conclusions and largely attempted to correct for uneven sample sizes by dividing estimates of genetic diversity by the sample size (Table 3 in Baker et al. (2017)). This method of correction is not appropriate (e.g., as genetic diversity and sample size have a nonlinear relationship, see Figure 2), and rarefaction is more typically used to compare estimates of genetic diversity among samples that differ in size (Petit, Mousadik, & Pons, 1998; Szpiech, Jakobsson, & Rosenberg, 2008). As an illustration, we obtained data for Baker et al.'s (2017) samples of bank voles for the mitochondrial locus ND4 (1,378 bp that had 60 variable sites) from DRYAD (https://doi.org/10.5061/dryad.j11s7) and calculated the number of haplotypes per sample, corrected for sample size using rarefaction implemented by ADZE (Szpiech et al., 2008). We also found that mitochondrial diversity (as measured by the number of haplotypes) is higher in contaminated than in uncontaminated sites (Figure 2), reinforcing Baker et al.'s (2017) conclusions; moreover, high diversity is apparent in the small (*n *=* *3) Glyboke Lake 2011 sample although these data were not included in the statistical comparison of population genetic diversity. Hence, the level of mitochondrial diversity is associated with the level of environmental radioactivity. But, do these data indicate a high mutation rate?

## 3 SAMPLING: UNCLEAR CHOICE OF CONTROL SITES

Several studies have quantified mitochondrial diversity (at a 291‐bp fragment of the control region and some adjacent tRNA) in bank voles inhabiting the CEZ and in uncontaminated sites in Ukraine (Matson, Rodgers, Chesser, & Baker, 2000; Meeks et al., 2007, 2009; Wickliffe et al., 2006): none of these studies concluded that there was a robust association between mutation rate and the level of environmental radionuclides. Rather, studies have highlighted the need for additional sampling (Matson et al., 2000; Wickliffe et al., 2006) or found mitochondrial diversity to be comparable between contaminated and uncontaminated sites that were located close to the CNPP (Meeks et al., 2007); moreover, genetic diversity was heterogeneous among samples of bank voles collected over a large area of Ukraine, with uncontaminated locations containing more unique haplotypes and a higher ratio of unique to total haplotypes (Table 1 in Meeks et al., 2009). Variation in mitochondrial diversity in bank voles has been explained by demographic and ecological processes, rather than exposure to environmental radionuclides (e.g., Meeks et al., 2009). These studies on bank vole mitochondrial genetic diversity were not addressed in detail by Baker et al. (2017) despite the conclusion by Meeks et al. (2009) that “*genetic diversity in radioactive regions of Ukraine is probably a function of natural geographic variation rather than increased mutational pressure from radiation exposure and underscore the importance of adequate geographic sampling*.”

**Table 1:** Heteroplasmy (Hp) estimates in each of the samples separately

Locality	Year	N_B_	N_Hp_	Proportion of Hp individuals	Hp sites/individual	Average frequency of heteroplasmies
Uncontaminated						
Nedanchychy	1998	11	11	0.727	1.182	0.134
Nedanchychy	2011	12	12	0.750	1.250	0.076
Nezamozhnya	1998	12	11 (12)	0.636 (0.667)	1.000 (3.083)	0.031
Nezamozhnya	2011	12	10	0.500	0.900	0.110
Oranoe	1998	15	12 (13)	0.583 (0.615)	0.833 (2.231)	0.083
Oranoe	2011	14	11	0.545	0.909	0.134
In total		76	67 (69)	0.627 (0.638)	1.015 (1.638)	0.078
Contaminated						
Glyboke Lake	1998	15	14	0.571	0.929	0.032
Glyboke Lake	2011	3	3	0.667	1.000	0.070
Red Forest	1998	20	18	0.722	1.167	0.055
Red Forest	2011	17	11	0.455	0.455	0.062
In total		55	46	0.609	0.913	0.050

For the average frequency of all heteroplasmies, frequency of the heteroplasmic allele was calculated separately for each heteroplasmy site within individuals. For the two samples with “outlier” individuals, Nezamozhnya and Oranoe (both 1998), values in parentheses are the estimates including the outliers. N_B_ represent the original sample sizes in Baker et al. (2017), while N_Hp_ is the amount of individuals used in the present heteroplasmy analysis.

The prior information about levels of mitochondrial diversity for bank vole populations within and around the CEZ is important when choosing sampling locations. Of the three control samples selected by Baker et al. (2017), Nedanchychy had notably less mitochondrial diversity than other bank vole samples from Ukraine (Meeks et al., 2007, 2009; Wickliffe et al., 2006): haplotype diversity (*h*) at Nedanchychy (*h *=* *0.19) is less than half that of the sample with the next lowest diversity (Ezyaslav, *h *=* *0.44) (Table 1 and Figure 2 in Meeks et al. (2009)). Reasons for low genetic diversity at Nedanchychy are not known, but highlight marked site‐specific variation in genetic diversity in wild bank vole populations. Although mitochondrial diversity at Nedanchychy appears atypical of bank vole populations in Ukraine, the impact of this sample on the conclusion about levels of mitochondrial diversity appears limited as the other reference samples are less diverse than the contaminated samples (Figure 2). Also, Meeks et al. (2007) sampled bank voles from six potential control (uncontaminated) locations that lie within about 40 km of the CNPP (Figure 1). A comparison of any these sites, rather than samples from distant areas, with contaminated samples would reduce the effect of historic demographic processed on patterns of genetic diversity. However, Baker et al. (2017) provide no reason for their analysis of the three more distant (>30 km from CNPP) controls rather than obtaining data from one or more of the uncontaminated sites (Stupnikovo, Krasnoye or Paryshev) that are closer (1–15 km) to the contaminated areas (see Figure 1). Locations within or close to the CEZ are the likely sources of the bank voles that recolonized the contaminated areas within the CEZ after the nuclear accident. As such, the sites Stupnikovo, Krasnoye or Paryshev might be the most appropriate controls for an assessment of the impact of environmental radionuclides on genetic diversity. Alternatively, use of these proximate sites might confound analyses of diversity due to ongoing dispersal of bank voles among contaminated and uncontaminated areas. Bank voles are fairly mobile and can move 1 km within a breeding season (Kozakiewicz, Chołuj, & Kozakiewicz, 2007). Choice of appropriate control sites is an important issue that, here, is complicated by a combination of prior information about variation in mitochondrial diversity in potential samples and a lack of knowledge about the population dynamics of wild bank voles.

## 4 BANK VOLE POPULATION DYNAMICS

The possible influence of population history on spatial patterns of mitochondrial diversity should be reconsidered. Baker et al. (2017) imply that their data are inconsistent with the hypothesis of recolonization explaining the observed high mitochondrial diversity in contaminated sites because the surrounding areas (potential sources) have lower mitochondrial diversity. However, an area recolonized by several, genetically different, sources could exhibit an increase in genetic diversity (as discussed by (Matson et al., 2000)). The uncontaminated areas to the east of Chernobyl (Nedanchychy and Nezamozhynya) are genetically different from a distant control site (Korostychev) that is south of Chernobyl (Meeks et al., 2009). Also, bank voles from uncontaminated sites Nedanchychy, Krasnove and Paryshev (Figure 1), as well as the contaminated sites within CEZ, have unique (not found at any other site) mitochondrial haplotypes (Meeks et al., 2007, 2009; Wickliffe et al., 2006). Indeed, there were no shared mitochondrial haplotypes among the CEZ and control regions (Figure 1 in Baker et al. (2017)). This pattern of genetic diversity indicates that gene flow among CEZ and control sites is limited, but it does not rule out recolonization of the CEZ from several surrounding source sites as other controls areas were not analysed by Baker et al. (2017) (Figure 1). As bank vole populations within and around the CEZ are genetically different, recolonization processes remain a possible reason for the high mitochondrial diversity of bank voles inhabiting the CEZ. This highlights the difficulties in associating a signature of genetic variation to the effects of ionizing radiation rather than other demographic population processes, an issue also pointed out by Baker et al. (2017).

A strength of the analysis by Baker et al. (2017) is their data from 1998 and 2011. The 13‐year difference between sampling periods is comparable to the time interval between the accident (1986) and the first time point (1998) and represents about 26 generations (of exposure to radionuclides). One corollary of the mutagenic effects of exposure to environmental radionuclides is that mutations accumulate with time. By contrast, no temporal change in mitochondrial diversity at the Red Forest (contaminated) sample was apparent (Tables 3 and 4 in Baker et al. (2017), also see Figure 2), although there were greater nucleotide differences between temporal samples at the contaminated sites, but small sample size prevented any statistical inference (Table S4 in Baker et al. (2017)). A lack of temporal effect weakens the argument that exposure to low‐dose radionuclides simply increases mutation. One implication is that most of the mitochondrial diversity (via mutation) may have accumulated at some point to affect the 1998 sample, but not subsequently. For example, most of the fallout from the Chernobyl accident was iodine‐131. Exposure to ^131^I conceivably might have had some initial, but not contemporary, impact on wildlife as this isotope dissipated rapidly (half‐life of 8 days). Initial exposure to radiation can trigger a suite of cellular effects that persist for some time (Mothersill & Seymour, 2006). Exposure to ^131^I is associated with elevated incidence of human thyroid cancers (Cardis et al., 2006), but its long‐term effects on wildlife are not known. Other principal radionuclides (^90^S, ^137^Cs and ^239^Pu—see *Introduction*) within the CEZ are more persistent, and their effects are less likely to have dissipated recently. We might speculate that bank voles exhibit some adaptive response to radionuclide contamination, for example, via improved antioxidative measures and/or DNA repair that prevents further accumulation of mutations. Fibroblasts of bank voles from the CEZ exhibit greater antioxidative capacity than do bank vole fibroblasts from control areas near Kiev (V. Mustonen, J. Kesäniemi, A. Lavrinienko, E. Tukalenko, T. Mappes, P. C. Watts, & J. Jurvansuu, unpublished results). We could also argue that any temporal genetic pattern in these bank vole data is confounded by stochastic recolonization of the CEZ from different source populations. With this in mind, Wickliffe et al. (2006) found marked fluctuations in mitochondrial diversity (haplotype diversity *h* varied from 0.67 to 0.82) among samples of bank voles from the contaminated Red Forest site over a three‐year period (1998–2001). The discussion above does not exclude a role for mutation, but highlights that other processes can explain the contemporary pattern of genetic diversity in bank voles within and around the CEZ.

## 5 HETEROPLASMY AS A MARKER OF MUTATION?

Inferring mutation rate from population genetic diversity itself is complicated by processes that determine whether (or not) a mutation is incorporated into the population at a detectable frequency: the probability that a mutation is retained within a population, for example, depends on strength of selection, population size, recombination (e.g., for nuclear DNA) and sample size. A complementary analysis of mutation could focus on mutations occurring within individuals independent of demography. One solution is to quantify heteroplasmy, the occurrence of more than one mitochondrial haplotype within an individual (Li, Schröder, Ni, Madea, & Stoneking, 2015; Li et al., 2010). Heteroplasmy may be caused by paternal transmission of mitochondrial DNA or reflect mutations produced by DNA replication errors, inefficient DNA repair or oxidative damage (Kmiec, Woloszynska, & Janska, 2006): an increase in heteroplasmy therefore could be a potential signal of exposure to mutagens. Heteroplasmy has been explored as a biomarker of exposure to environmental radioactivity in bank voles from the CEZ, where exposure to radionuclides elicited a nonsignificant increase in heteroplasmy (Wickliffe, Chesser, Rodgers, & Baker, 2002). Next‐generation sequencing (NGS) is well suited for detecting heteroplasmy as the high depth of coverage that can be readily achieved when (re)sequencing a small genome (*e.g*., mitochondrial DNA) allows for robust detection of intra‐individual polymorphisms (Li et al., 2010; Tang & Huang, 2010; Wachsmuth, Hübner, Li, Madea, & Stoneking, 2016); for example, at a 1,000× coverage, a heteroplasmy occurring at 1% frequency is expected to be visible in about 10 reads, a signal that should be distinct from the numbers of mismatches derived from a ~0.1% error rate associated with Illumina HiSeq2000 chemistry (Glenn, 2011). Baker et al.'s (2017) NGS data present a powerful opportunity to examine the potential association between exposure to environmental radionuclides and heteroplasmy as (i) heteroplasmy is common in muscle (Li et al., 2015), the source of bank vole genetic material, and (ii) the authors achieved high depth of coverage (average coverage = 3,974, range = 64–7,841; table S1 in Baker et al. (2017)) over most mitochondrial genomes.

We obtained Baker et al.'s (2017) NGS data from NCBI's sequence read archive (https://www.ncbi.nlm.nih.gov/sra/, project accession SRX2515630). Only 122 of the 131 bank vole samples described by Baker et al. (2017) were archived. Sample information was not provided with the raw read data, so we assigned a putative origin by matching the count of raw reads in each file to the read data information provided in table S1 by Baker et al. (2017). Potential adaptors and poor quality reads were removed from the raw data using TRIMMOMATIC v.0.35 (Bolger, Lohse, & Usadel, 2014) (minimum length = 90, quality score = 20, sliding window size = 5). Paired reads were mapped to a bank vole mitochondrial genome (GenBank accession NC_024538) using BOWTIE2 v.2.2.9 (Langmead & Salzberg, 2012) (mapping options: ‐D 5 ‐R 1 ‐N 0 ‐L 22 ‐i S,0,2.50), as the mitochondrial reference for mapping by Baker et al. (2017) was not public at the time of analysis. Mapping data were sorted and converted to a MPILEUP file using SAMtools v.1.4 (http://samtools.github.io/hts-specs/SAMv1.pdf). Potential heteroplasmic sites were called using VARSCAN v.2.3.9 (Koboldt et al., 2012) on the basis of a minimum read frequency of 1%, but only when a minimum read depth of 500 was achieved and when at least 10% of the reads mapped to the alternate strand (to reduce numbers of false‐positive sites arising from PCR artefacts (Scarcelli et al., 2016)). Variable sites were called between positions 220 and 15,793 of the reference genome due to low coverage at the beginning and end of the reference. This analysis allowed us to quantify heteroplasmy on the basis of (i) whether an individual contained at least one heteroplasmy or not and (ii) the total number of heteroplasmic sites found within an individual's mitochondrial genome. As seven samples had low sequencing coverage across the entire mitochondrial genome, we had a final sample of 115 individuals (*n *=* *46 and 69 from contaminated and uncontaminated sites, respectively) for analysis of heteroplasmy in bank voles (Table 1).

As it is in many other animals (Kmiec et al., 2006), heteroplasmy appears to be common in bank voles as we identified 72 (63%) individuals with at least one heteroplasmic site, represented by 28 (61%) and 44 (64%) individuals from the contaminated and uncontaminated sites, respectively (Table 1). Most individuals with heteroplasmy contained only 1 or 2 heteroplasmic sites (1 site = 60%; 2 sites = 22%; ≥3 sites = 18% of data). The average number of heteroplasmic sites per individual was 0.91 in the contaminated samples and 1.02 in the uncontaminated samples (or 1.64 with the whole data set of uncontaminated samples, see below for discussion about outlier individuals). Additionally, the average frequency of heteroplasmy within the variable sites was similar in contaminated and uncontaminated areas (Table 1). Heteroplasmies were detected in bank vole mitochondria at 131 positions (at 90 positions without the two outlier individuals). Two bank voles contained apparently many heteroplasmic sites, both of which were taken from control areas in 1998: one individual from Nezamozhnya with 26 sites and one individual from Oranoe with 19 sites. Read mapping for these two individuals was visually inspected in TABLET v.1.14.11.07 (Milne et al., 2009). The potential heteroplasmic sites were scattered around the mitochondrial genome and represented by pairs of reads that had different insert sizes, implying that the heteroplasmy detection was not simply an artefact of PCR bias. Nonetheless, statistical analyses of variation in heteroplasmy with radionuclide contamination were made with and without the two “outlier” samples (note that both are from uncontaminated sites in 1998). We estimated whether levels of heteroplasmy differed between contaminated and uncontaminated sites using the generalized linear mixed model (GLMM) implemented by the GLMER function in LME4 (Bates, Mächler, Bolker, & Walker, 2015) run in R v.3.1.1 (R Development Core Team, 2014). Models examining whether an individual contained a heteroplasmy (Hp) or not (Proportion of Hp individuals) were treated with a binomial error distribution, while models using the number of heteroplasmies present within each individual (Hp sites/individual) used a Poisson distribution. Contamination (yes, no) and year (1998, 2011) were represented as fixed factors, and the five sample sites (Figure 1) were included as a random factor (See full results in Table S1). With all data (*n *=* *115), the proportion of individuals with a heteroplasmy was lower in the contaminated sites and also in 2011, although neither effect was significant (*p *=* *.63 and .31, respectively; Table 2). The numbers of heteroplasmies in individuals were significantly lower in contaminated sites and in 2011 (*p *<* *.001 for both predictors; Table 2). The qualitative pattern of lower heteroplasmy in the contaminated areas and a possible temporal reduction in heteroplasmy (between 1998 and 2011) remains when the two outlier individuals are removed, but with no significant predictor for either measure (presence/absence or count) of heteroplasmy (Table 2). Hence, analysis of Baker et al.'s (2017) NGS data yield no evidence that exposure to environmental radiation is associated with the level of heteroplasmy. Qualitatively, bank voles inhabiting the CEZ have lower levels of heteroplasmy and exhibit a decrease in the level of heteroplasmy between 1998 and 2011 in the Red Forest (Tables 1 and 2). Neither of these spatial nor temporal patterns is an expected consequence of a simple, positive association between chronic exposure to environmental radionuclides and the rate of mutation.

**Table 2:** Summarized results of generalized linear mixed models (GLMM) testing the effects of contamination and sampling year on the levels of heteroplasmy (Hp) of bank voles from contaminated and uncontaminated sites

	*n *= 115		*n *= 113	
Proportion of Hp individuals				
Effect	Estimate	*p*	Estimate	*p*
Intercept	0.765	.018	0.697	.033
Contaminated site	−0.196	.625	−0.145	.719
Year 2011	−0.406	.308	−0.353	.377
Hp sites/individual				
Effect	Estimate	*p*	Estimate	*p*
Intercept	0.777	<.001	0.094	.534
Contaminated site	−0.697	<.001	−0.137	.493
Year 2011	−0.727	<.001	−0.167	.401

GLMM was run with all available individuals (*n* = 115) and a reduced data set with the two outlier individuals from uncontaminated sites removed (*n* = 113).

## 6 DISCUSSION AND CONCLUSIONS

Understanding the biological effects of exposure to low‐dose radiation is an important issue given that numerous human activities have left substantial amounts of radionuclides in the environment (Lourenço et al., 2016): reports of accelerated mutation rate have clear policy implications. While a high rate of mutation is characteristic of diverse taxa affected by Chernobyl fallout (Geras'kin, Fesenko, & Alexakhin, 2008), the specific responses to radionuclide exposure vary between taxa (Møller & Mousseau, 2015) and mammals are comparatively understudied. Application of NGS techniques represents a much needed scientific advance for studies of wildlife inhabiting the CEZ. However, sequence data for whole mitochondrial genomes (from Baker et al., 2017) are also consistent with the results of previous studies of bank vole mitochondrial diversity at the control region with the results being explained by processes other than mutation (e.g., Matson et al., 2000; Meeks et al., 2007, 2009; Wickliffe et al., 2006). Analysis of heteroplasmy in bank voles offers high power to detect low‐frequency intra‐individual mutations and can circumvent the uncertainty associated with inferring mutation from populations whose demographic histories are unknown. A lack of association between heteroplasmy and contamination by environmental radionuclides is important as occurrence of low‐frequency, intra‐individual mutations is presumably needed to generate the “raw material” for mutations that are later visible as “population genetic diversity.” A recent meta‐analysis has revealed an association between mutation rate and environmental radiation exposure in many species from Chernobyl (Møller & Mousseau, 2015). However, given our discussion about sampling, bank vole population history and heteroplasmy, we suggest that in addition to the report of high mitochondrial diversity in samples of bank voles inhabiting the CEZ, further studies are needed to demonstrate an accelerated mutation rate in this species.

## References

[eva12578-bib-0001] Baker, R. J. , Dickins, B. , Wickliffe, J. K. , Khan, F. A. A. , Gaschak, S. , Makova, K. D. , & Phillips, C. D. (2017). Elevated mitochondrial genome variation after 50 generations of radiation exposure in a wild rodent. Evolutionary Applications., 10(8), 784–791. https://doi.org/10.1111/eva.12475 29151870 10.1111/eva.12475 PMC5680428

[eva12578-bib-0002] Bates, D. , Mächler, M. , Bolker, B. M. , & Walker, S. C. (2015). Fitting linear mixed‐effects models using lme4. Journal of Statistical Software, 67, 1–48. https://doi.org/10.18637/jss.v067.i01

[eva12578-bib-0003] Bolger, A. M. , Lohse, M. , & Usadel, B. (2014). Trimmomatic: A flexible trimmer for Illumina sequence data. Bioinformatics, 30, 2114–2120. https://doi.org/10.1093/bioinformatics/btu170 24695404 10.1093/bioinformatics/btu170 PMC4103590

[eva12578-bib-0004] Boratyński, Z. , Lehmann, P. , Mappes, T. , Mousseau, T. A. , & Møller, A. P. (2014). Increased radiation from Chernobyl decreases the expression of red colouration in natural populations of bank voles (*Myodes glareolus*). Scientific Reports, 4, 7141 https://doi.org/10.1038/srep07141 25413373 10.1038/srep07141 PMC5382704

[eva12578-bib-0005] Cardis, E. , Howe, G. , Ron, E. , Bebeshko, V. , Bogdanova, T. , Bouville, A. , … Zvonova, I. (2006). Cancer consequences of the Chernobyl accident: 20 years on. Journal of Radiological Protection, 26, 127–140. https://doi.org/10.1088/0952-4746/26/2/001 16738412 10.1088/0952-4746/26/2/001

[eva12578-bib-0006] Chesser, R. K. , Sugg, D. W. , Lomakin, M. D. , Van den Bussche, R. A , DeWoody, J. A , Jagoe, C. H. , … Baker, R. J. (2000). Concentrations and dose rate estimates of (134,137)cesium and (90)strontium in small mammals at Chornobyl, Ukraine. Environmental Toxicology and Chemistry, 19, 305–312. https://doi.org/10.1002/etc.5620190209

[eva12578-bib-0007] Geras'kin, S. A. , Fesenko, S. V. , & Alexakhin, R. M. (2008). Effects of non‐human species irradiation after the Chernobyl NPP accident. Environment International, 34, 880–897. https://doi.org/10.1016/j.envint.2007.12.012 18234336 10.1016/j.envint.2007.12.012

[eva12578-bib-0008] Glenn, T. C. (2011). Field guide to next‐generation DNA sequencers. Molecular Ecology Resources, 11, 759–769. https://doi.org/10.1111/j.1755-0998.2011.03024.x 21592312 10.1111/j.1755-0998.2011.03024.x

[eva12578-bib-0009] Kahle, D. H. , & Wickham, H. (2016). ggmap: Spatial Visualization with ggplot2. The R Journal, 5, 144–161.

[eva12578-bib-0010] Kmiec, B. , Woloszynska, M. , & Janska, H. (2006). Heteroplasmy as a common state of mitochondrial genetic information in plants and animals. Current Genetics, 50, 149–159. https://doi.org/10.1007/s00294-006-0082-1 16763846 10.1007/s00294-006-0082-1

[eva12578-bib-0011] Koboldt, D. C. , Zhang, Q. , Larson, D. E. , Shen, D. , McLellan, M. D. , Lin, L. , … Wilson, R. K. (2012). VarScan 2: Somatic mutation and copy number alteration discovery in cancer by exome sequencing. Genome Research, 22, 568–576. https://doi.org/10.1101/gr.129684.111 22300766 10.1101/gr.129684.111 PMC3290792

[eva12578-bib-0012] Kozakiewicz, M. , Chołuj, A. , & Kozakiewicz, A. (2007). Long‐distance movements of individuals in a free‐living bank vole population: An important element of male breeding strategy. Acta Theriologica, 52, 339–348. https://doi.org/10.1007/bf03194231

[eva12578-bib-0013] Langmead, B. , & Salzberg, S. L. (2012). Fast gapped‐read alignment with Bowtie 2. Nature Methods, 9, 357–359. https://doi.org/10.1038/nmeth.1923 22388286 10.1038/nmeth.1923 PMC3322381

[eva12578-bib-0014] Lehmann, P. , Boratyński, Z. , Mappes, T. , Mousseau, T. A. , & Møller, A. P. (2016). Fitness costs of increased cataract frequency and cumulative radiation dose in natural mammalian populations from Chernobyl. Scientific Reports, 6, 19974 https://doi.org/10.1038/srep19974 26814168 10.1038/srep19974 PMC4728484

[eva12578-bib-0015] Li, M. , Schönberg, A. , Schaefer, M. , Schroeder, R. , Nasidze, I. , & Stoneking, M. (2010). Detecting heteroplasmy from high‐throughput sequencing of complete human mitochondrial DNA genomes. American Journal of Human Genetics, 87, 237–249. https://doi.org/10.1016/j.ajhg.2010.07.014 20696290 10.1016/j.ajhg.2010.07.014 PMC2917713

[eva12578-bib-0016] Li, M. , Schröder, R. , Ni, S. , Madea, B. , & Stoneking, M. (2015). Extensive tissue‐related and allele‐related mtDNA heteroplasmy suggests positive selection for somatic mutations. Proceedings of the National Academy of Sciences of the United States of America, 112, 2491–2496. https://doi.org/10.1073/pnas.1419651112 25675502 10.1073/pnas.1419651112 PMC4345623

[eva12578-bib-0017] Lourenço, J. , Mendo, S. , & Pereira, R. (2016). Radioactively contaminated areas: Bioindicator species and biomarkers of effect in an early warning scheme for a preliminary risk assessment. Journal of Hazardous Materials, 317, 503–542. https://doi.org/10.1016/j.jhazmat.2016.06.020 27343869 10.1016/j.jhazmat.2016.06.020

[eva12578-bib-0018] Matson, C. W. , Rodgers, B. E. , Chesser, R. K. , & Baker, R. J. (2000). Genetic Diversity of *Clethrionomys Glareolus* Populations From Highly Contaminated Sites in the Chornobyl Region, Ukraine. Environmental Toxicology and Chemistry, 19, 2130–2135. https://doi.org/10.1002/etc.5620190824

[eva12578-bib-0019] Meeks, H. N. , Chesser, R. K. , Rodgers, B. E. , Gaschak, S. , & Baker, R. J. (2009). Understanding the genetic consequences of environmental toxicant exposure: Chernobyl as a model system. Environmental Toxicology and Chemistry/SETAC, 28, 1982–1994. https://doi.org/10.1897/08-578.1 10.1897/08-578.1 19388794

[eva12578-bib-0020] Meeks, H. N. , Wickliffe, J. K. , Hoofer, S. R. , Chesser, R. K. , Rodgers, B. E. , & Baker, R. J. (2007). Mitochondrial control region variation in bank voles (*Clethrionomys glareolus*) is not related to Chernobyl radiation exposure. Environmental Toxicology and Chemistry/SETAC, 26, 361–369. https://doi.org/10.1897/06-346r.1 10.1897/06-346r.1 17713225

[eva12578-bib-0021] Milne, I. , Bayer, M. , Cardle, L. , Shaw, P. , Stephen, G. , Wright, F. , & Marshall, D. (2009). Tablet‐next generation sequence assembly visualization. Bioinformatics, 26, 401–402. https://doi.org/10.1093/bioinformatics/btp666 19965881 10.1093/bioinformatics/btp666 PMC2815658

[eva12578-bib-0022] Møller, A. P. , & Mousseau, T. A . (2006). Biological consequences of Chernobyl: 20 years on. Trends in Ecology and Evolution, 21, 200–207. https://doi.org/10.1016/j.tree.2006.01.008 16701086 10.1016/j.tree.2006.01.008

[eva12578-bib-0023] Møller, A. P. , & Mousseau, T. A. (2015). Strong effects of ionizing radiation from Chernobyl on mutation rates. Scientific Reports, 5, 8363 https://doi.org/10.1038/srep08363 25666381 10.1038/srep08363 PMC4322348

[eva12578-bib-0024] Mothersill, C. , & Seymour, C. (2006). Radiation‐induced bystander effects: Evidence for an adaptive response to low dose exposures? Dose‐Response, 4, 283–290. https://doi.org/10.2203/dose-response.06-111.mothersill 18648593 10.2203/dose-response.06-111.Mothersill PMC2477684

[eva12578-bib-0025] Mousseau, T. , & Møller, A. (2012). Chernobyl and Fukushima: Differences and Similarities, a Biological Perspective. Transactions of the American Nuclear Society, 107, 200–203.

[eva12578-bib-0026] Petit, R. J. , Mousadik, A. E. L. , & Pons, O. (1998). Identifying populations for conservation on the basis of genetic markers. Conservation Biology, 12, 844–855. https://doi.org/10.1111/j.1523-1739.1998.96489.x

[eva12578-bib-0027] R Development Core Team . (2014). R: A Language and Environment for Statistical Computing. Vienna, Austria: R Foundation for Statistical Computing Retrieved from http://www.R-project.org/

[eva12578-bib-0028] Rodgers, B. E. , & Baker, R. J. (2000). Frequencies of micronuclei in bank voles from zones of high radiation at Chornobyl, Ukraine. Environmental Toxicology and Chemistry, 19, 1644–1648. https://doi.org/10.1002/etc.5620190623

[eva12578-bib-0029] Rodgers, B. E. , Wickliffe, J. K. , Phillips, C. J. , Chesser, R. K. , & Baker, R. J. (2001). Experimental exposure of naive bank voles (*Clethrionomys glareolus*) to the Chornobyl, Ukraine, environment: A Test of radioresistance. Environmental Toxicology and Chemistry, 20, 1936–1941. https://doi.org/10.1002/etc.5620200911 11521819

[eva12578-bib-0030] Scarcelli, N. , Mariac, C. , Couvreur, T. L. P. , Faye, A. , Richard, D. , Sabot, F. , … Vigouroux, Y. (2016). Intra‐individual polymorphism in chloroplasts from NGS data: Where does it come from and how to handle it? Molecular Ecology Resources, 16, 434–445. https://doi.org/10.1111/1755-0998.12462 26388536 10.1111/1755-0998.12462

[eva12578-bib-0031] Szpiech, Z. A. , Jakobsson, M. , & Rosenberg, N. A. (2008). ADZE: A rarefaction approach for counting alleles private to combinations of populations. Bioinformatics, 24, 2498–2504. https://doi.org/10.1093/bioinformatics/btn478 18779233 10.1093/bioinformatics/btn478 PMC2732282

[eva12578-bib-0032] Tang, S. , & Huang, T. (2010). Characterization of mitochondrial DNA heteroplasmy using a parallel sequencing system. BioTechniques, 48, 287–296. https://doi.org/10.2144/000113389 20569205 10.2144/000113389

[eva12578-bib-0033] Wachsmuth, M. , Hübner, A. , Li, M. , Madea, B. , & Stoneking, M. (2016). Age‐Related and Heteroplasmy‐Related Variation in Human mtDNA Copy Number. PLoS Genetics, 12, e1005939 https://doi.org/10.1371/journal.pgen.1005939 26978189 10.1371/journal.pgen.1005939 PMC4792396

[eva12578-bib-0034] Wickliffe, J. K. , Chesser, R. K. , Rodgers, B. E. , & Baker, R. J. (2002). Assessing the genotoxicity of chronic environmental irradiation by using mitochondrial DNA heteroplasmy in the bank vole (*Clethrionomys glareolus*) at Chornobyl, Ukraine. Environmental Toxicology and Chemistry, 21, 1249–1254. https://doi.org/10.1002/etc.5620210619 12069310

[eva12578-bib-0035] Wickliffe, J. K. , Dunina‐Barkovskaya, Y. V , Gaschak, S. P. , Rodgers, B. E. , Chesser, R. K. , Bondarkov, M. , & Baker, R. J. (2006). Variation in mitochondrial DNA control region haplotypes in populations of the bank vole, *Clethrionomys glareolus*, living in the Chernobyl environment, Ukraine. Environmental Toxicology and Chemistry/SETAC, 25, 503–508. https://doi.org/10.1897/05-327r.1 10.1897/05-327r.1 16519312

