# Supplementary material for: Analysis of heteroplasmy in bank voles inhabiting the Chernobyl exclusion zone: A commentary on Baker et al. (2017) “Elevated mitochondrial genome variation after 50 generations of radiation exposure in a wild rodent.”
Source: Evol Appl. 2018 Jan 17;11(5):820–6. doi: 10.1111/eva.12578 (PMC5978973; doi:10.1111/eva.12578)
Supplement: Supplementary file 1 [file EVA-11-820-s001.docx]

**Supplemental Information**

**Table 1.** Full results of generalized linear mixed model (GLMM) testing the effects of contamination and sampling year on the levels of heteroplasmy (Hp) of Ukrainian bank voles. Models were run with all available individuals (n=115) and a reduced data set with the two outlier individuals form uncontaminated sites removed (n=113).

|  |  |  |  |  |
| --- | --- | --- | --- | --- |
| **Proportion of Hp individuals** *n=115* | | |  |  |
| Effect | Estimate | SE | z | P |
| Intercept | 0.765 | 0.322 | 2.374 | 0.018 |
| Contaminated sites | -0.196 | 0.401 | -0.490 | 0.625 |
| Year 2011 | -0.406 | 0.398 | -1.020 | 0.308 |
|  |  |  |  |  |
| **Hp sites/ individual** *n=115* | |  |  |  |
| Effect | Estimate | SE | z | P |
| Intercept | 0.777 | 0.112 | 6.929 | <0.001 |
| Contaminated sites | -0.697 | 0.185 | -3.762 | <0.001 |
| Year 2011 | -0.727 | 0.183 | -3.983 | <0.001 |
|  |  |  |  |  |
| **Proportion of Hp individuals** *n=113* | | |  |  |
| Effect | Estimate | SE | z | P |
| Intercept | 0.697 | 0.327 | 2.133 | 0.033 |
| Contaminated sites | -0.145 | 0.403 | -0.359 | 0.719 |
| Year 2011 | -0.353 | 0.400 | -0.883 | 0.377 |
|  |  |  |  |  |
| **Hp sites/ individual** *n=113* | |  |  |  |
| Effect | Estimate | SE | z | P |
| Intercept | 0.094 | 0.151 | 0.621 | 0.534 |
| Contaminated sites | -0.137 | 0.200 | -0.685 | 0.493 |
| Year 2011 | -0.167 | 0.200 | -0.839 | 0.401 |
